# Postoperative C-Reactive Protein Resolution Patterns in Patients With and Without Postoperative Atrial Fibrillation After Cardiac Surgery

**DOI:** 10.1093/icvts/ivag179

**Published:** 2026-06-29

**Authors:** Melina Heine, Felix Ulbrich, Samuel Justice, Babak Saravi, Simon A Amacher, Julian Hubrich, Leonard Simeth, Charles N Serhan, Jochen D Muehlschlegel, Jakob Wollborn

**Affiliations:** Department of Anesthesiology, Mass General Brigham, Brigham and Women’s Hospital, Harvard Medical School, Boston, MA 02115, United States; Department of Anesthesiology and Critical Care, Medical Center, University of Freiburg, 79106 Freiburg im Breisgau, Germany; Department of Anesthesiology, Mass General Brigham, Brigham and Women’s Hospital, Harvard Medical School, Boston, MA 02115, United States; Department of Oral, Maxillofacial and Facial Plastic Surgery, Medical Faculty and University Hospital Düsseldorf, Heinrich-Heine-University, 40225 Düsseldorf, Germany; Department of Anesthesiology and Critical Care, Medical Center, University of Freiburg, 79106 Freiburg im Breisgau, Germany; Department of Anesthesiology, Mass General Brigham, Brigham and Women’s Hospital, Harvard Medical School, Boston, MA 02115, United States; Department of Anesthesiology, Mass General Brigham, Brigham and Women’s Hospital, Harvard Medical School, Boston, MA 02115, United States; Department of Anesthesiology, Mass General Brigham, Brigham and Women’s Hospital, Harvard Medical School, Boston, MA 02115, United States; Department of Anesthesiology and Critical Care Medicine, Johns Hopkins University School of Medicine, Baltimore, MD 21287, United States; Department of Anesthesiology, Mass General Brigham, Brigham and Women’s Hospital, Harvard Medical School, Boston, MA 02115, United States

**Keywords:** POAF, cardiac surgery, inflammation, CRP, interleukins, postoperative complications

## Abstract

**Objectives:**

Postoperative atrial fibrillation is common after cardiac surgery. Effective preventative measures are lacking, partly due to an incomplete understanding of its pathogenesis. Inflammation is a major contributor to postoperative atrial fibrillation development. This study aims to further characterize the association between systemic inflammation and postoperative atrial fibrillation.

**Methods:**

A secondary analysis was conducted on a single-center cohort of patients undergoing cardiac surgery. The original study enrolled 405 cardiac surgery patients. After exclusion of patients with a history of persistent or permanent atrial fibrillation, patients undergoing heart transplantation, and patients with incomplete interleukin measurements, 319 patients were included in the present analysis. Postoperative atrial fibrillation was identified through chart review and was defined as at least 1 AF episode lasting more than 30 s, as documented by telemetry or a 12-lead electrocardiogram during the patient’s initial hospitalization. Perioperative levels of interleukins and C-reactive protein were measured. The decline in C-reactive protein levels between postoperative days 3 and 10 was modeled using a linear regression. The association with postoperative atrial fibrillation and other outcomes after cardiac surgery was assessed using logistic and Tobit regression models.

**Results:**

Of the 319 patients included in the study, 51% developed postoperative atrial fibrillation, most commonly on postoperative days 2 and 3. Cardiopulmonary bypass and intraoperative ventilation time were significantly associated with an increased risk of postoperative atrial fibrillation. Patients with postoperative atrial fibrillation had longer intensive care unit stays and longer times to extubation. C-reactive protein levels peaked on postoperative day 3 and declined more slowly in postoperative atrial fibrillation patients. A slower C-reactive protein decline was independently associated with postoperative atrial fibrillation (odds ratio: 1.71, *P* = .046), prolonged postoperative intensive care unit length of stay, and time to extubation. Interleukin-10 levels were significantly higher in patients with postoperative atrial fibrillation (mean ratio: 1.28, *P* < .001).

**Conclusions:**

Postoperative atrial fibrillation is a prevalent complication after cardiac surgery and correlates with a slower postoperative C-reactive protein decline. This study suggests that postoperative atrial fibrillation is associated with delayed resolution of postoperative inflammation.

**Trial registration number:**

German Clinical Trials Registry (DRKS No. 00017057), Date of registration April 5, 2019, www.drks.de.

## Graphical abstract

**Figure:**
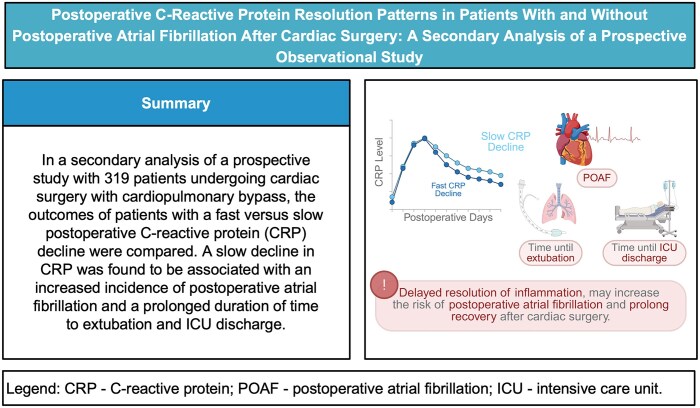

## INTRODUCTION

**Figure 1.**
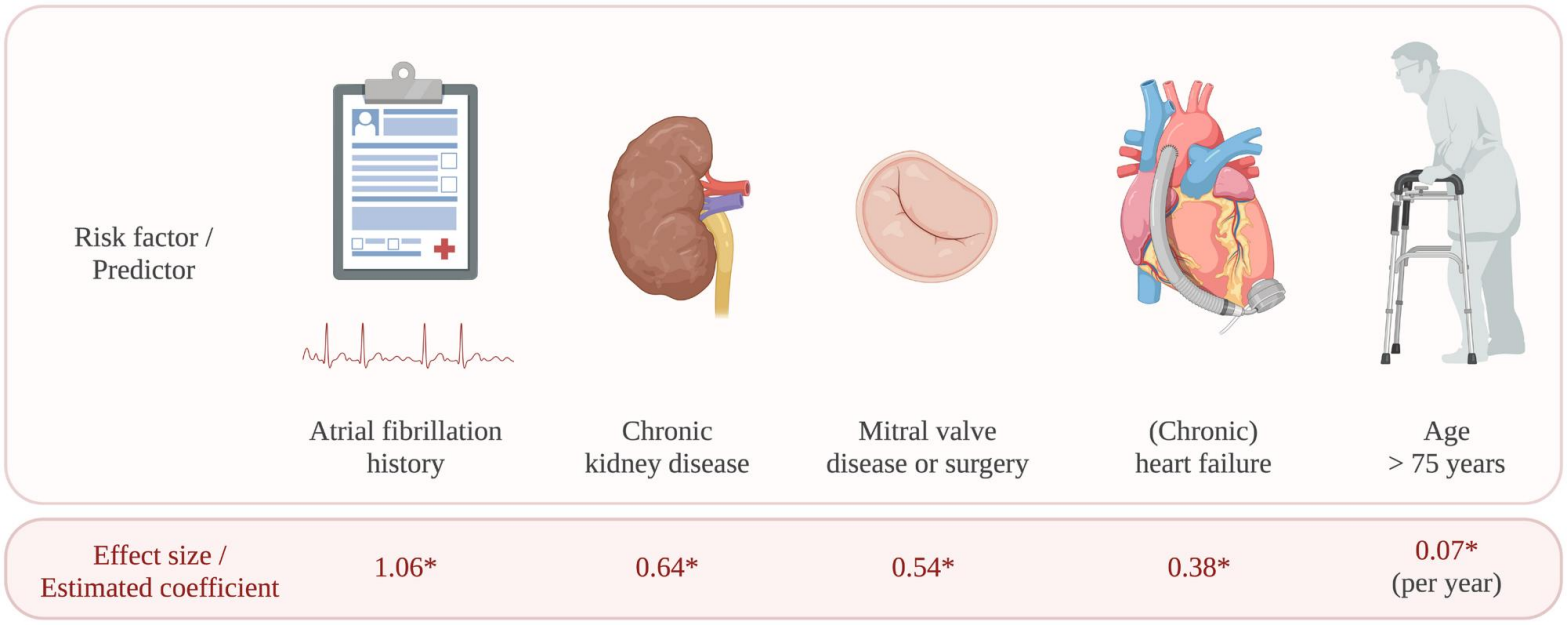
Overview of Baseline POAF Risk Factors. *The estimated coefficients for postoperative atrial fibrillation (POAF) risk factors were obtained from O’Brien et al.^1^ For predictors reported in multiple studies, combined effects were calculated as sample-size-weighted mean coefficients. The coefficient of each study was weighted based on its sample size, divided by the total sample size of all studies that reported that particular predictor

Postoperative atrial fibrillation (POAF) is the most common complication in patients undergoing cardiac surgery with the use of cardiopulmonary bypass. Its incidence ranges from 30 to 50%.^1^ POAF most frequently occurs between postoperative days (POD) 2 and 4, with the highest incidence on POD 2.^2^ POAF is associated with longer stays in the intensive care unit, prolonged hospitalization, and complicated recovery. It has been linked to several adverse perioperative outcomes, including an increased risk for thromboembolic events (e.g., stroke) and an increased perioperative mortality rate.^3^ Various risk factors have been identified for the development of POAF after cardiac surgery (**Figure 1**).^1^^,^^2^

Both local and systemic inflammation have been identified as key drivers in the development of POAF. A variety of factors have been found to contribute to the inflammatory response observed in patients undergoing cardiac surgery, including surgical trauma to the heart, accumulation of pericardial fluid, ischemia-reperfusion injury and the systemic pro-inflammatory effects associated with cardiopulmonary bypass (CPB).^4^^,^^5^ The combination of structural alterations and electrophysiologic changes in the atria of the heart can be induced by inflammation. Perioperative inflammation of the atrial tissue temporarily disrupts the heart’s conduction system, favoring reentry circuits, whereas sustained inflammation promotes fibrotic changes.^1^^,^^6^ Several established inflammatory markers have been associated with the occurrence of atrial fibrillation, among them C-reactive protein (CRP) and interleukins (ILs).^7–11^

Our study aims to further characterize the association between systemic inflammation, including the dynamic trajectory of CRP and POAF, in a cohort of patients undergoing cardiac surgery. There is an emerging consensus that timely inflammatory resolution is critical for recovery.^12^ Inflammatory resolution is defined as an active process characterized by the biosynthesis of mediators that restore tissue integrity and function.^13^ We hypothesize that prolonged inflammation after cardiac surgery, as indicated by a slower decline in CRP, is associated with an elevated risk for POAF development. Impaired or delayed inflammatory resolution may contribute to sustained inflammation, which in turn promotes arrhythmogenic remodeling of the heart and increases the heart’s susceptibility to POAF.

## METHODS

### Design and setting

**Figure 2.**
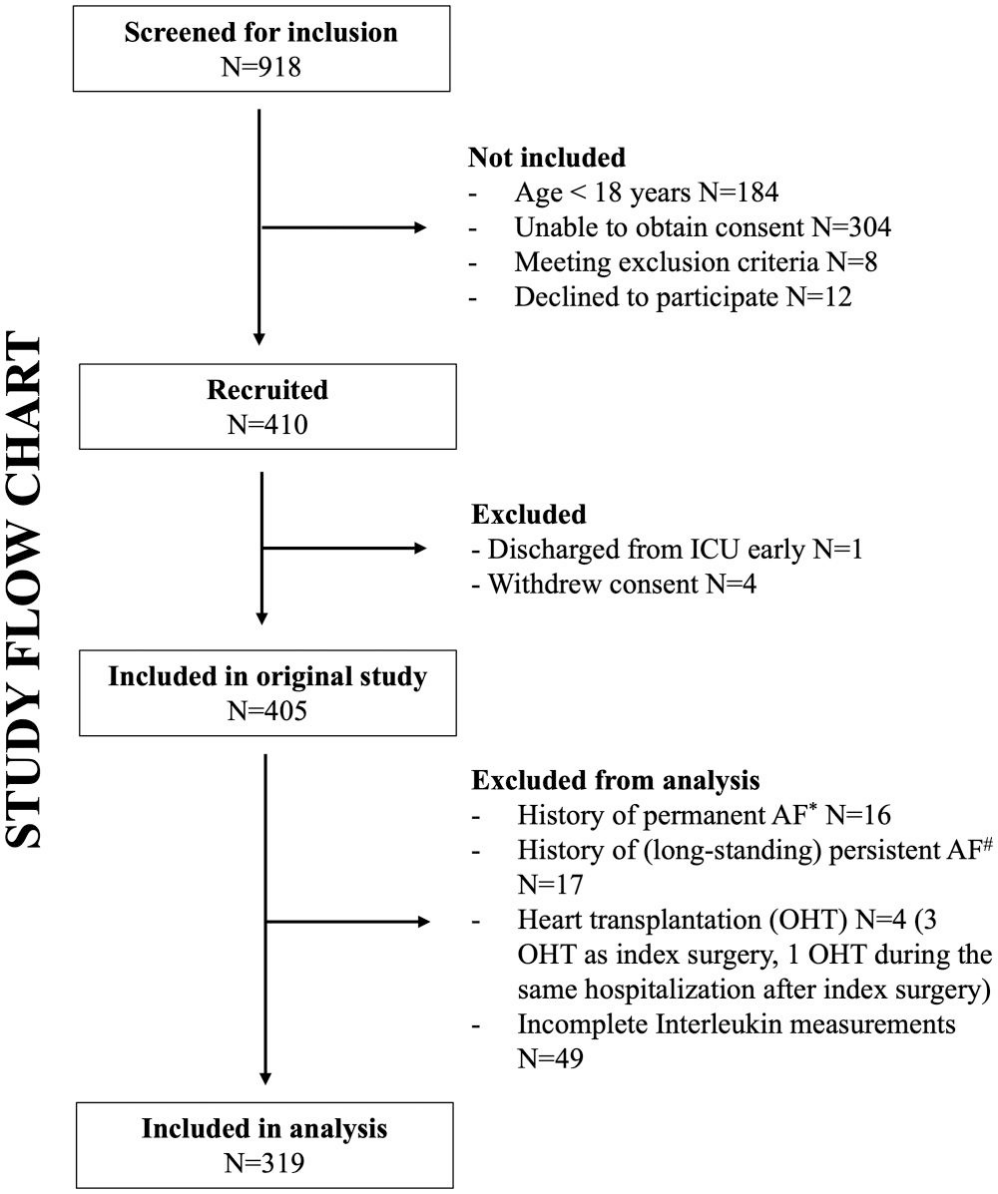
Study Flow Chart. Persistent^#^ and permanent* atrial fibrillation according to the 2024 ESC/EACTS guidelines for the management of atrial fibrillation.^22^ Abbreviation: ICU, intensive care unit; AF, atrial fibrillation; OHT, orthotopic heart transplantation

This is a secondary analysis from a prospective, single-center cohort study of 405 patients who underwent open-heart surgery with cardiopulmonary bypass at the University Medical Center Freiburg between May 2019 and October 2020.^14^ The study was approved by the institutional review board (Freiburg, EK-Nr. 405/18) and subsequently registered in the German Clinical Trials Register (DRKS No. 00017057). Of the 918 patients who were screened for inclusion, 410 were recruited (**Figure 2**). Written consent was obtained from the patients or their legal guardians. The study was conducted and reported according to the STROBE guidelines.

### Study population

**Figure 3.**
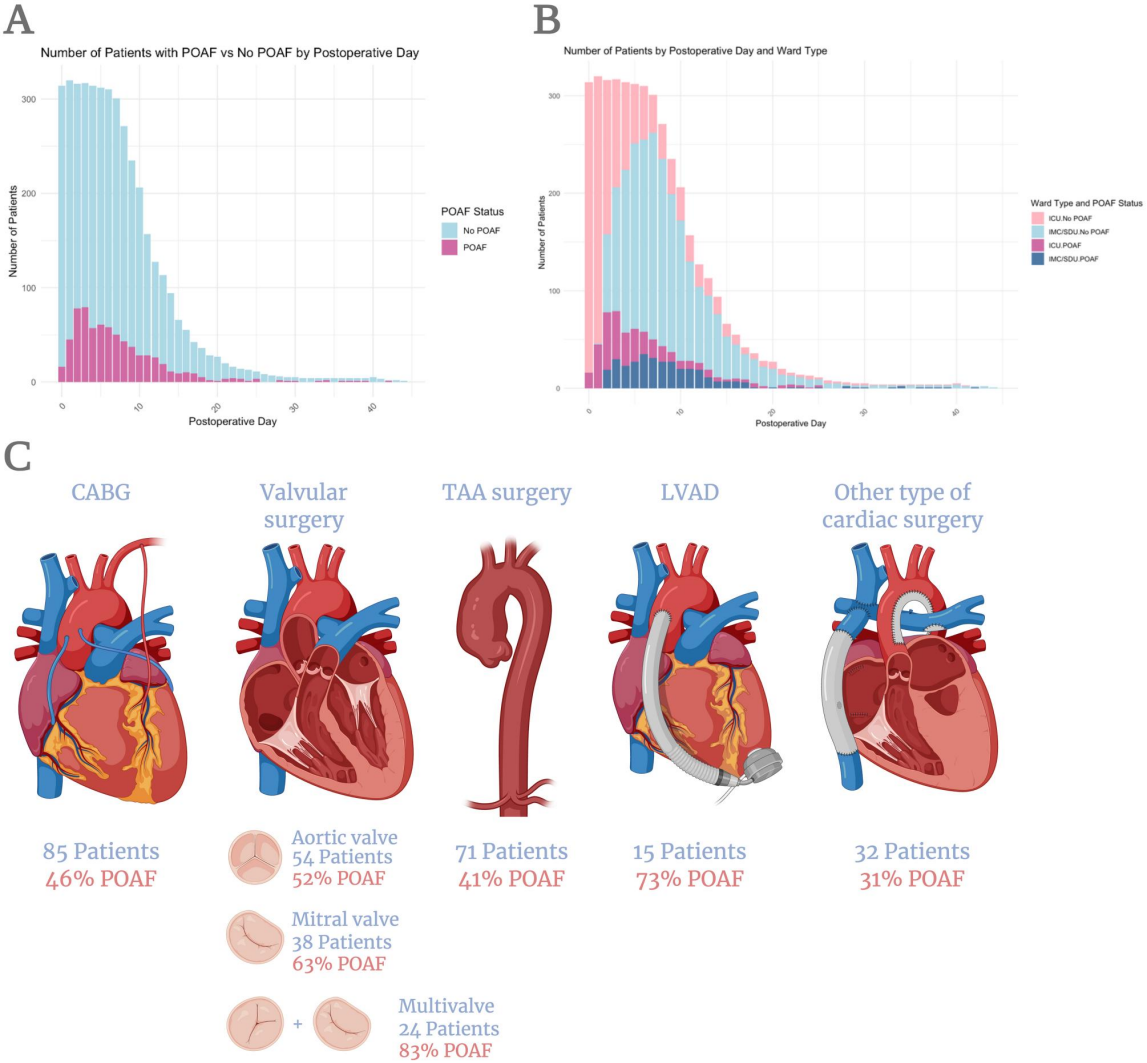
Temporal POAF Distribution and POAF Incidence in Surgical Groups. (A) Number of patients with and without POAF across postoperative days. (B) The stacked bars show the cohort’s temporal and spatial characteristics during the postoperative period, along with their POAF status. (C) POAF incidence across surgical subtypes (blue: number of patients per group; red: proportion (%) Who Developed POAF). Abbreviations: CABG, coronary artery bypass grafting; ICU, intensive care unit; IMC, intermediate care unit; LVAD, left ventricular assist device; POAF, postoperative atrial fibrillation; SDU, step-down unit; TAA, thoracic aortic aneurysm

The original study was designed to characterize the capillary leak syndrome (CLS) after cardiac surgery with CPB. Inclusion and exclusion criteria were defined based on the study’s original aim.^14^ For this secondary analysis, we further excluded patients with longstanding AF, those undergoing heart transplantation, and those with incomplete IL measurements (**Figure 2**). The remaining 319 patients were assigned to surgical groups (CABG, aortic valve surgery, mitral valve surgery, multi-valve surgery, thoracic aortic surgery, LVAD implantation, and other types of surgery) based on the type of cardiac surgery performed (see **Figure 3C**). In cases of combined procedures, the more extensive procedure determined the assignment, e.g., a patient undergoing CABG and aortic valve surgery was assigned to the aortic valve surgical group.

### Clinical care

The study did not change existing clinical care pathways in accordance with institutional guidelines. Patients underwent cardiac surgery with CPB and were admitted to the intensive care unit (ICU) after the procedure. As soon as considered appropriate, patients were transferred to the step-down unit (SDU).

### Study measurements

In addition to routine blood draws, blood samples were collected preoperatively after general anesthesia was induced, postoperatively after the procedure ended, and daily while the patients were in the ICU to allow for interleukin measurements. All available data were used to detect POAF in the cohort. Patients needed evidence of at least 1 episode of AF lasting longer than 30 s or evidence of AF in a 12-lead ECG after surgery during the index hospitalization to be classified as POAF patients. CRP levels were extracted from routine laboratory charts, providing measurements throughout the entire hospital stay. Blood samples were analyzed using the BD Cytometric Bead Array Human Inflammatory Cytokine Kit to measure IL-6, -8, and -10 levels in all 319 patients.

### Statistical analysis

Analysis was performed using the R statistical software. Descriptive statistics were used to summarize baseline characteristics and intra- and postoperative factors, as appropriate. Continuous variables were compared using a *t*-test or the Wilcoxon rank-sum test, depending on distribution, while categorical variables were compared using the Chi-square test or Fisher’s exact test, as appropriate. Multivariable logistic regression was used to evaluate the association between CPB duration and intraoperative ventilation time with the occurrence of POAF. We adjusted for 5 established POAF risk factors (**Figure 1**).^1^ The mitral valve disease or surgery risk factor included patients who underwent mitral valve surgery and patients with any echocardiographic evidence of mitral valve stenosis or insufficiency.

Missing data were minimal. All available data were included for the characteristics reported in **Table 1**. Patients with incomplete interleukin measurements were excluded prior to analysis. In the CRP slope analysis, 20 patients could not be assigned to a group due to an insufficient number of postoperative CRP measurements between POD 3 and 10. No imputation was performed.

**Table 1.** Patient Demographics, Clinical Characteristics, Intra- and Postoperative Factors

	**Total** (*n* = 319)
Demographics	
Age (mean ± SD), years	61.8 ± 14.1
Sex, no. (%)	
Male	238 (74.6)
Clinical characteristics	
Congestive heart failure, no. (%)	60 (18.81)
History of paroxysmal AF or atrial flutter, no. (%)	71 (22.26)
CKD, no. (%)	40 (12.54)
Antiarrhythmics before admission, no. (%)	161 (50.47)
Left atrial enlargement (LA diameter), no. (%)	123 (38.56)
Intraoperative factors	
EuroScore I (mean ± SD)	5 ± 4.17
CPB time (median ± IQR), min	126 (95.3-178.8)
Cross clamp time (median ± IQR), min	92 (65-123)
Ventilation time (median ± IQR), hours	7 (6-8)
Occurrence of intraoperative AF, no. (%)	19 (5.96)
Postoperative characteristics	
Occurrence of postoperative AF, no. (%)	161 (50.47)
Length of ICU stay (LOS, median ± IQR), days	4 (2-6.5)
LOS step-down unit (median ± IQR), days	8 (6-11)
Need for cardiac reoperation, no. (%)	44 (13.79)
Duration until extubation (median ± IQR), hours	8 (5.5-16)

Abbreviations: AF, atrial fibrillation; CKD, chronic kidney disease; CPB, cardiopulmonary bypass; IQR, interquartile range; LOS, length of stay; POAF, postoperative atrial fibrillation; SD, standard deviation.

The slope of the CRP decline between postoperative days (POD) 3 and 10 was derived for each patient using a linear model and expressed as the change in CRP concentration (mg/l) per day. Postoperative day 3 was chosen as the starting point because CRP levels usually begin to decline at this time. POD 10 marked the point at which CRP levels plateaued postoperatively or showed only minimal further decrease. Patients were then assigned to 2 groups based on the median value of the calculated CRP slope. Patients with more negative slopes, indicating a faster reduction in postoperative CRP levels over time, were assigned to the fast CRP decline group. Conversely, patients with less negative slopes, indicating a slower reduction in CRP levels, were assigned to the slow CRP decline group.

Logistic regression, adjusted for the 5 previously defined POAF risk factors, was applied to evaluate the association between group assignment and POAF occurrence. Logistic regression was also used to analyze the relationship between CRP group assignment and the occurrence of acute kidney injury (AKI), adjusting for age, sex, transfusion of packed red blood cells, POD 1 catecholamine dependence, obesity, and surgical group (see Ref. 14).

For the secondary outcomes of ICU discharge and postoperative extubation in the slow versus fast CRP decline groups, time-to-event analyses were performed using a competing risks framework, with death treated as a competing event. Cumulative incidence functions were compared using Gray’s test. Kaplan-Meier curves were used for visualization purposes, censoring deaths.

To account for the non-negativity and potential censoring of the IL-10 data (given the test assays’ lower detection limit of 3.3 pg/ml) along with repeated patient measures taken throughout the perioperative period, a mixed-effects Tobit regression model with a gamma link function was utilized to assess the association between POAF and IL-10 levels, adjusted for the 5 POAF risk factors. The results of this model were summarized using mean ratios, which are interpreted analogously to an odds ratio.

## RESULTS

### Baseline characteristics

Most of the patients underwent CABG or thoracic aortic aneurysm surgery. The mean (± standard deviation, SD) age of this cohort was 62 ± 14 years, and 74.6% of the patients were male. The mean EuroScore I was 5 ± 4 (**Table 1**).

Among this cardiac surgical cohort, 51% of the patients developed POAF during their hospitalization. The highest incidence occurred on the second (24.7%) and third (24.9%) POD (**Figure 3A and B**). Although the number of patients in each surgical group varied, we observed the highest POAF incidence within the multivalve (83%) group (**Figure 3C**).

### Outcomes

CPB duration and intraoperative ventilation time were significantly associated with an increased risk of developing POAF. Our multivariable logistic regression analysis revealed that each additional minute of CPB time was associated with a 1% increase in the odds of developing POAF (OR: 1.01, 95% CI: 1.00-1.01, *P* = .012). Similar findings were observed in a logistic regression examining the association between intraoperative ventilation time and POAF, showing a 16% increase in the odds of POAF for each additional hour (OR: 1.16, 95% CI: 1.00-1.35, *P* = .048). Both logistic regressions were adjusted for 5 POAF risk factors.

### Inflammatory markers

**Figure 4.**
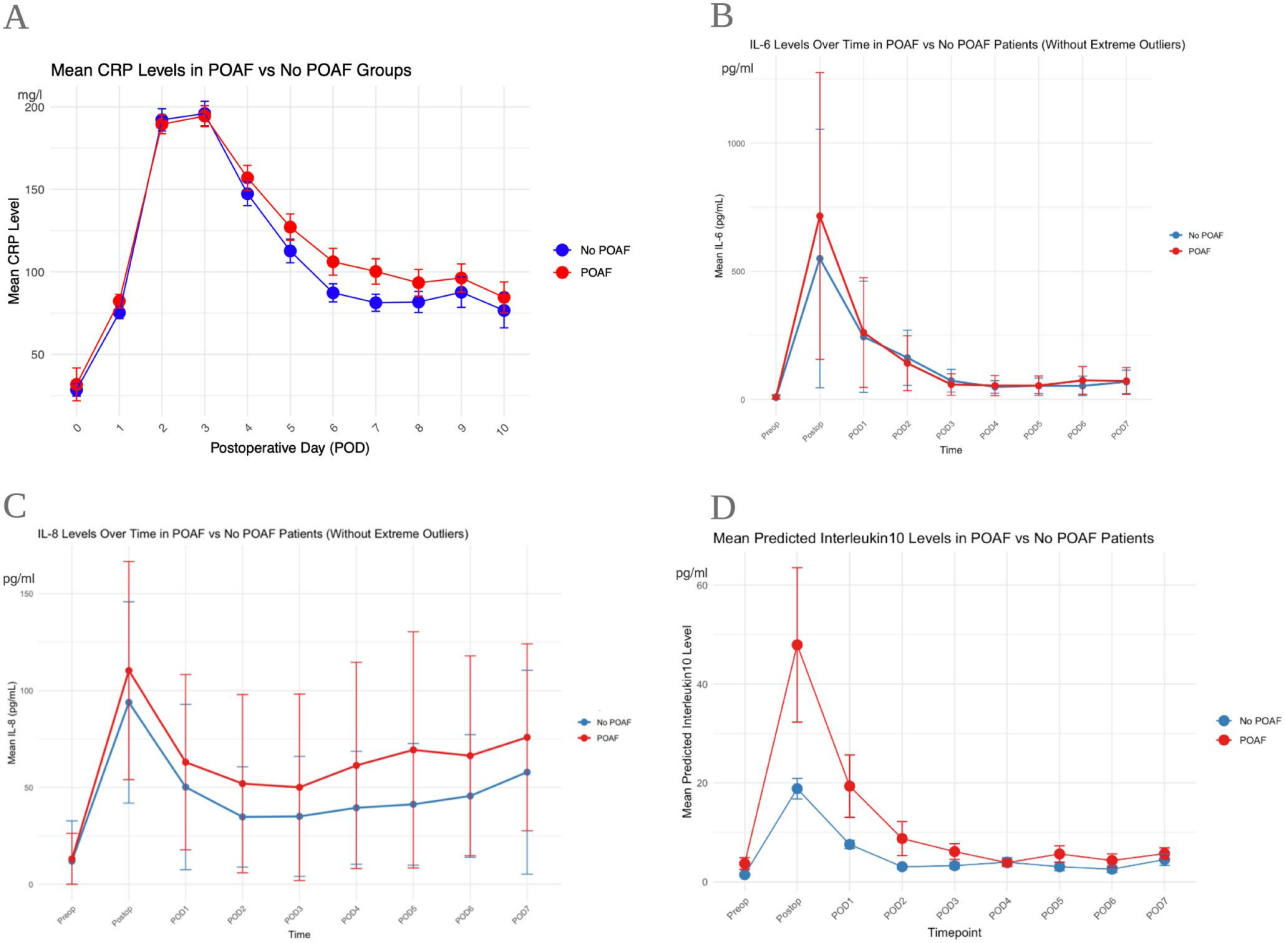
Postoperative CRP and Perioperative IL Dynamics (Proinflammatory: IL-6 and IL-8; anti-Inflammatory: IL-10). (A) Postoperative CRP Levels (mean ± SD) in patients with (red) and without (blue) POAF. Analogously perioperative IL-6 (B) and IL-8 Level (C) in POAF (red) and non-POAF (blue) patients (mean ± SD). (D) Predictions for IL-10 level in POAF and non-POAF patients (mean ± SD; predictions from Tobit regression). Abbreviations: CRP, C-reactive protein; IL, interleukin; POAF, postoperative atrial fibrillation; POD, postoperative day; SD, standard deviation

We observed an increase in CRP levels after surgery, with peak levels typically occurring on postoperative day 3 (**Figure 4A**). The decline of the postoperative CRP between POD3 and POD10 was significantly slower in the POAF patients (mean ± SD: −18.8 ± 1.5 vs −25.6 ±1.47 mg/l per day, *P* = .001). **Figures 4B-D** show, respectively, the mean levels of Interleukin-6, -8, and -10 in POAF and non-POAF patients during the perioperative period, with the highest levels observed immediately after surgery (timepoint “postop”). After adjusting for POAF risk factors (**Figure 1**), POAF patients exhibited significantly higher IL-10 levels throughout the perioperative period compared to non-POAF patients (mean ratio (MR): 1.28, 95% CI: 1.17-1.39, *P* < .001).

**Figure 5.**
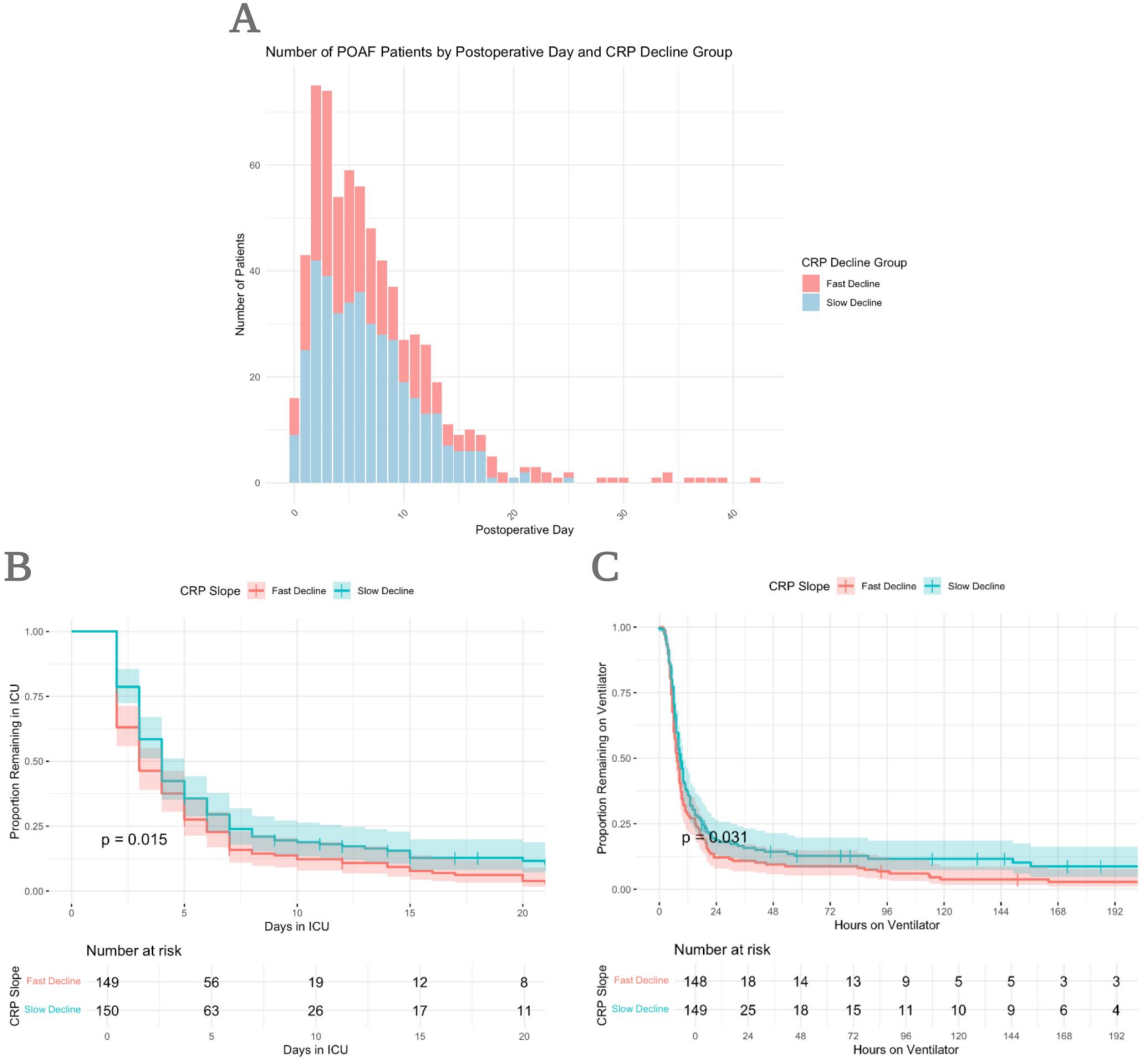
Temporal POAF Distribution (A) and Survival Analysis for ICU Stay (B) and Postoperative Ventilation Time (C) in the Slow and Fast CRP Decline Groups. Competing risks analyses accounting for death as a competing event confirmed these findings (see Results section). Abbreviations: CRP, C-reactive protein; ICU, intensive care unit; POAF; postoperative atrial fibrillation

Two groups were defined based on the median slope of the postoperative CRP decline between POD 3 and POD 10 using a linear model: a slow CRP decline group (*n* = 150) and a fast CRP decline group (*n* = 149). A significantly higher incidence of POAF was observed in the slow CRP decline group (59%) than in the fast decline group (45%, *P* = .01). The highest POAF incidence occurred on POD 2 and 3 in both groups (**Figure 5A**). Logistic regression adjusted for common POAF risk factors (**Figure 1**) showed a higher odds ratio for POAF development in the slow decline group (OR: 1.71, 95% CI: 1.01-2.88, *P* = .046). The association between slow CRP decline and POAF remained directionally consistent after exclusion of patients with any history of atrial fibrillation or flutter (OR: 1.67, 95% CI: 0.96-2.94, *P* = .07).

Furthermore, the slope of the postoperative CRP decline was also associated with other secondary outcomes after cardiac surgery: Unadjusted Kaplan-Meier survival analysis showed that patients in the slow decline group had a longer time to postoperative extubation (*P* = .031) and stayed in the ICU longer (*P* = .015) (**Figure 5B and C**). These results were confirmed in competing risk analysis, which demonstrated a significantly lower incidence of ICU discharge in the slow CRP decline group compared to the fast decline group (Gray’s test, *P* = .003). A similar pattern was observed for postoperative extubation (*P* = .007). No significant difference in the incidence of acute kidney injury (AKI) was observed between the 2 groups.

To further explore CRP as a graded predictor in a sensitivity analysis, patients were divided into quartiles based on their postoperative CRP slope. Logistic regression using group 1 (fastest decline) as reference, adjusted for the same risk factors, showed a trend toward higher odds of POAF with slower CRP decline, without reaching statistical significance.

## DISCUSSION

The key findings of this secondary analysis of a prospective, observational clinical study are as follows: The postoperative CRP decline was slower in patients with POAF compared to those without. Additionally, perioperative IL-10 levels were significantly higher in patients who developed POAF. Notably, a slower postoperative CRP decline was associated with a higher risk of developing POAF, as well as prolonged time to postoperative extubation and ICU length of stay.

Inflammation has emerged as a critical underlying mechanism of POAF development.^1^^,^^2^ In the context of cardiac surgery, the induction of inflammation is multifactorial, arising from both direct trauma to the pericardium and the heart itself as well as a systemic inflammatory response primarily attributable to the CPB.^4^^,^^5^ Studies suggest that the risk of developing AF following heart surgery is significantly higher with CPB than without, likely due to a more extensive inflammatory response.^11^^,^^15^^,^^16^

In line with prior studies, we observed a coincidence of peak CRP levels and the highest incidence of POAF on POD 2 and 3.^4^ Postoperative levels of IL-6, IL-8, and IL-10 were found to be elevated in the POAF patients as compared to patients without POAF, which aligns with the results of previous studies.^9^

This study supports the key role of inflammation in POAF pathogenesis and provides new insights by analyzing the dynamics of CRP levels, not just absolute values. After defining 2 groups based on the median postoperative CRP slope between POD 3 and 10, we demonstrated that a slower postoperative CRP decline is independently associated with a higher POAF risk. Furthermore, a slower CRP decline also correlated with a longer time to extubation and a longer ICU length of stay. This observation suggests a potential role for CRP as a marker for complicated postoperative recovery. In addition to confirming elevated perioperative IL-10 levels in patients with POAF, this study introduces an association between POAF and delayed inflammatory resolution, rather than differences in peak inflammation alone. Sensitivity analysis dividing patients into quartiles further suggests a graded relationship. The reasons why inflammatory markers decline more rapidly in some patients than in others remain incompletely understood. A number of perioperative factors may contribute to a persistent inflammatory state. These might include the extent of surgical trauma, CPB-related systemic inflammatory activation and the burden of preexisting comorbidities.^5^^,^^17^^,^^18^

Inflammatory resolution, once thought to be a passive process, is now known to be an active biosynthetic process involving the production of specialized mediators that promote the resolution of inflammation.^13^ These specialized pro-resolving mediators (SPMs), such as resolvins, have recently gained attention for their potential to modulate heart diseases, including AF.^19^ Inflammatory resolution is involved in terminating the inflammatory response while restoring tissue integrity and function.^13^ Interindividual differences in endogenous pro-resolving pathways might have the potential to influence the rate of resolution of the inflammatory response. The observed difference in postoperative CRP trajectories may be partially attributed to variability in the production or activity of SPMs.^12^^,^^20^ Furthermore, preliminary animal studies suggest that promoting resolution pathways may reduce arrhythmogenesis; however, the clinical application of this finding is still in its infancy.^21^ A better understanding of the influence of inflammation and inflammatory resolution could lead to novel targets for POAF risk stratification, prevention and treatment.

This study has several strengths. First, the relatively large and heterogeneous patient cohort, encompassing a broad spectrum of cardiac surgeries and illness severity, enhances the generalizability of the findings and allowed for a robust analysis of inflammatory markers and their association with POAF. Second, multiple diagnostic modalities were used to identify POAF. This enhanced diagnostic accuracy and likely contributed to a higher rate of POAF in our cohort. Third, inflammatory markers were systematically monitored. CRP values were available throughout the entire hospital stay. To our knowledge, this is the first study to examine not only absolute levels of inflammatory markers, but also the dynamic course of CRP, specifically the rate of its postoperative decline, and its relationship to POAF and clinical outcomes.

However, the study is not without limitations. As a secondary analysis, it relies on the conduct and design of the primary study as well as existing medical records for the diagnosis of POAF, which may have introduced variability due to differences in documentation quality. The patient population was predominantly male (75%), and a higher-than-usual rate of POAF (51%) was observed. These factors may limit the generalizability of the results to broader patient populations, particularly female patients or lower-risk surgical candidates. Paroxysmal AF is a recognized risk factor for POAF; therefore, it may be a potential source of residual confounding despite statistical adjustment. However, in a sensitivity analysis that excluded these patients, the association between slower CRP decline and POAF exhibited a similar trend but did not reach statistical significance (*P* = .07), likely attributable to the reduced sample size. An analytic limitation exists in the dichotomization of the postoperative CRP decline into “slow” versus “fast” CRP decline via a median split. While this approach facilitates interpretability, it also sacrifices statistical power. Further analysis would benefit from modeling CRP decline as a continuous variable to better capture the nuances of inflammatory resolution. CRP is a nonspecific inflammatory marker. Within the cohort, 10 of 319 patients (3%) experienced a documented perioperative infection. While this proportion was negligible and is unlikely to have substantially altered overall CRP trajectories, a confounding effect of infection on inflammatory patterns cannot be entirely excluded.

Given the sample size of 319 patients, the number of covariates in the logistic regression was limited to 5 well-established POAF risk factors to avoid overfitting and to preserve model stability. However, the exclusion of variables that are known to influence both inflammation and arrhythmia risk may introduce confounders. The number of available blood samples for IL measurements decreased after POD 1 due to patient transfers out of the ICU, introducing attrition bias and limiting the analysis of inflammatory trajectories beyond the early postoperative phase. In contrast, CRP levels were routinely measured daily until hospital discharge.

Due to the observational design, a definitive causal relationship between inflammatory changes and POAF onset cannot be established. Because most POAF episodes occur early after surgery, reverse causation, where POAF influences CRP levels, cannot be ruled out. Nevertheless, these findings generate hypotheses that may serve as the foundation for future trials aiming to modulate postoperative inflammation after cardiac surgery.

## CONCLUSION

In conclusion, this study highlights the role of inflammation in the development of POAF after cardiac surgery. A slower postoperative decrease in CRP was found to be independently associated with an increased risk of postoperative atrial fibrillation. Our results support previous research linking inflammation to arrhythmogenesis and introduce the concept of delayed inflammatory resolution as a potential contributor to POAF. By examining CRP dynamics, rather than just peak levels, we provide new insights into the timing and progression of inflammation and its relationship with POAF development. Additionally, our study emphasizes the potential of inflammatory markers in identifying high-risk patients who may benefit from preventive strategies, including antiarrhythmic treatment or early intervention. Despite the limitations of a retrospective design, our findings provide valuable insights that could guide future research on targeted anti-inflammatory or novel pro-resolving therapies, improving risk stratification and enhancing patient outcomes.

## Data Availability

The data underlying this article will be shared on reasonable request to the corresponding author.

## STORED DATA AND BIOBANK

Biological samples and clinical data were stored for research purposes in accordance with the Declaration of Taipei.

## ABBREVIATIONS

AF: atrial fibrillation
AKI: acute kidney injury
CABG: coronary artery bypass grafting
CHF: congestive heart failure
CI: confidence interval
CKD: chronic kidney disease
CLS: capillary leak syndrome
CPB: cardiopulmonary bypass
CRP: C-reactive protein
ECG: electrocardiogram
HIV: human immunodeficiency virus
ICU: intensive care unit
IL: interleukin
IMC: intermediate care unit
LVAD: left ventricular assist device
MR: mean ration
OR: odds ratio
POAF: postoperative atrial fibrillation
POD: postoperative day
SDU: step-down unit

## References

[1] O’Brien B , Burrage PS, Ngai JY, et al Society of Cardiovascular Anesthesiologists/European Association of Cardiothoracic Anaesthetists Practice Advisory for the management of perioperative atrial fibrillation in patients undergoing cardiac surgery. J Cardiothorac Vasc Anesth. 2019;33:12-26. 10.1053/j.jvca.2018.09.039 30591178

[2] Echahidi N , Pibarot P, O’Hara G, Mathieu P. Mechanisms, prevention, and treatment of atrial fibrillation after cardiac surgery. J Am Coll Cardiol. 2008;51:793-801. 10.1016/j.jacc.2007.10.043 18294562

[3] Caldonazo T , Kirov H, Rahouma M, et al; POAF-MA Group. Atrial fibrillation after cardiac surgery: a systematic review and meta-analysis. J Thorac Cardiovasc Surg. 2023;165:94-103.e24. 10.1016/j.jtcvs.2021.03.077 33952399

[4] Gaudino M , Di Franco A, Rong LQ, Piccini J, Mack M. Postoperative atrial fibrillation: from mechanisms to treatment. Eur Heart J. 2023;44:1020-1039. 10.1093/eurheartj/ehad019 36721960 PMC10226752

[5] Millar JE , Fanning JP, McDonald CI, McAuley DF, Fraser JF. The inflammatory response to extracorporeal membrane oxygenation (ECMO): a review of the pathophysiology. Crit Care. 2016;20:387. 10.1186/s13054-016-1570-4 27890016 PMC5125043

[6] Ishii Y , Schuessler RB, Gaynor SL, et al Inflammation of atrium after cardiac surgery is associated with inhomogeneity of atrial conduction and atrial fibrillation. Circulation. 2005;111:2881-2888. 10.1161/circulationaha.104.475194 15927979

[7] Zhou X , Dudley SC. Evidence for inflammation as a driver of atrial fibrillation. Front Cardiovasc Med. 2020;7:62. 10.3389/fcvm.2020.00062 32411723 PMC7201086

[8] Galea R , Cardillo MT, Caroli A, et al Inflammation and C-reactive protein in atrial fibrillation: cause or effect? Tex Heart Inst J. 2014;41:461-468. 10.14503/THIJ-13-3466 25425976 PMC4189345

[9] Weymann A , Popov AF, Sabashnikov A, et al Baseline and postoperative levels of C-reactive protein and interleukins as inflammatory predictors of atrial fibrillation following cardiac surgery: a systematic review and meta-analysis. Kardiol Pol. 2018;76:440-451. 10.5603/KP.a2017.0242 29354906

[10] Saskin H , Serhan Ozcan K, Yilmaz S. High preoperative monocyte count/high-density lipoprotein ratio is associated with postoperative atrial fibrillation and mortality in coronary artery bypass grafting. Interact CardioVasc Thorac Surg. 2017;24:395-401. 10.1093/icvts/ivw376 28040764

[11] Jakubova M , Mitro P, Stancak B, et al The occurrence of postoperative atrial fibrillation according to different surgical settings in cardiac surgery patients. Interact CardioVasc Thorac Surg. 2012;15:1007-1012. 10.1093/icvts/ivs361 22927177 PMC3501296

[12] Fredman G , Serhan CN. Specialized pro-resolving mediators in vascular inflammation and atherosclerotic cardiovascular disease. Nat Rev Cardiol. 2024;21:808-823. 10.1038/s41569-023-00984-x 38216693 PMC12863063

[13] Serhan CN. Pro-resolving lipid mediators are leads for resolution physiology. Nature. 2014;510:92-101. 10.1038/nature13479 24899309 PMC4263681

[14] Wollborn J , Zhang Z, Gaa J, et al Angiopoietin-2 is associated with capillary leak and predicts complications after cardiac surgery. Ann Intensive Care. 2023;13:70. 10.1186/s13613-023-01165-2 37552379 PMC10409979

[15] Zhang P , Wang L, Zhai K, et al Off-pump versus on-pump redo coronary artery bypass grafting: a systematic review and meta-analysis. Perfusion. 2021;36:724-736. 10.1177/0267659120960310 33016239

[16] Taha A , Hjärpe A, Martinsson A, et al Cardiopulmonary bypass management and risk of new-onset atrial fibrillation after cardiac surgery. Interdiscip Cardiovasc Thorac Surg. 2023;37:ivad153. 10.1093/icvts/ivad153 37713475 PMC10533753

[17] Akintoye E , Sellke F, Marchioli R, Tavazzi L, Mozaffarian D. Factors associated with postoperative atrial fibrillation and other adverse events after cardiac surgery. J Thorac Cardiovasc Surg. 2018;155:242-251.e10. 10.1016/j.jtcvs.2017.07.063 28890081

[18] Olesen OJ , Vinding NE, Østergaard L, et al C-reactive protein after coronary artery bypass graft surgery and its relationship with postoperative atrial fibrillation. Europace. 2020;22:1182-1188. 10.1093/europace/euaa088 32623472

[19] Hiram R. Resolution-promoting autacoids demonstrate promising cardioprotective effects against heart diseases. Mol Biol Rep. 2022;49:5179-5197. 10.1007/s11033-022-07230-6 35142983 PMC9262808

[20] Szczuko M , Zawadzka K, Szczuko U, Rudak L, Pobłocki J. The significance and process of inflammation involving eicosapentaenoic and docosahexaenoic derivatives in Hashimoto’s disease. Nutrients. 2025;17:1715. 10.3390/nu17101715 40431455 PMC12113837

[21] Hiram R , Xiong F, Naud P, et al The inflammation-resolution promoting molecule resolvin-D1 prevents atrial proarrhythmic remodelling in experimental right heart disease. Cardiovasc Res. 2021;117:1776-1789. 10.1093/cvr/cvaa186 32866246 PMC8208753

[22] Van Gelder IC , Rienstra M, Bunting KV, et al; ESC Scientific Document Group. 2024 ESC guidelines for the management of atrial fibrillation developed in collaboration with the European Association for Cardio-Thoracic surgery (EACTS). Eur Heart J. 2024;45:3314-3414. 10.1093/eurheartj/ehae176 39210723

